# Warping Torsion in Sandwich Panels: Analyzing the Structural Behavior through Experimental and Numerical Studies

**DOI:** 10.3390/ma17020460

**Published:** 2024-01-18

**Authors:** Eric Man Pradhan, Jörg Lange

**Affiliations:** Institute for Steel Construction and Materials Mechanics, Technical University of Darmstadt, 64287 Darmstadt, Germany; info@stahlbau.tu-darmstadt.de

**Keywords:** sandwich panels, eccentrically loaded, warping torsion, structural behavior, rotation, stress analysis, parametric studies, experimental investigations, numerical model, finite element

## Abstract

Recently, there has been a growing interest in the use of sandwich panels that, beyond handling well-known bending stress, can withstand torsional stresses. This is particularly relevant for wall applications when the panels are equipped with photovoltaic or supplemental curtain wall modules. This research article presents a detailed exploration of the structural behavior of eccentrically loaded sandwich panels, with a specific focus on warping torsion. Experimental and numerical studies were conducted on polyisocyanurate (PU) core sandwich panels, commonly employed in building envelopes. These studies involved various dimensions and material properties, while omitting longitudinal joints. The experimental study provided essential insights and validated the numerical model in ANSYS. Enabling parametric variation, the numerical analysis extends the analysis beyond the experimental scope. Results revealed a high degree of correlation between experimental, numerical, and analytical solutions, regarding the rotation, as well as the normal and shear stress of the panel. Confirming the general applicability of warping torsion in sandwich panels with certain limitations, the study contributes valuable data for applications and design of eccentrically loaded sandwich panels, laying the foundation for potential engineering calculation methods.

## 1. Introduction

### 1.1. Motivation and Objective

**Figure 1:**
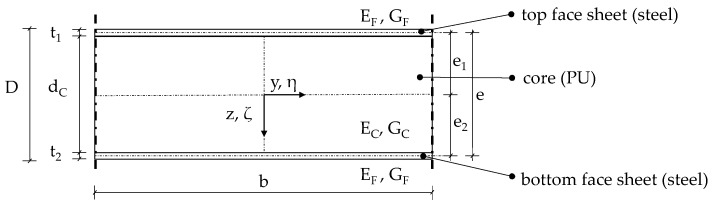
Definition of the sandwich panel cross-section (without joint geometry).

Sandwich panels, consisting of two thin steel face sheets with a shear-deformable polyisocyanurate (PU) or mineral wool core in between have become a well-established solution for cost-effective building envelopes, especially within the industrial building sector in Europe. They offer the benefits of a prefabricated lightweight component, delivering excellent thermal insulation and sealing performance. In Germany alone, around 20 million square meters of sandwich panels, employed for cladding and roofing applications, were manufactured and installed annually. Figure 1 illustrates a typical cross-section with notations indicating the dimensions and material properties.

**Figure 2:**
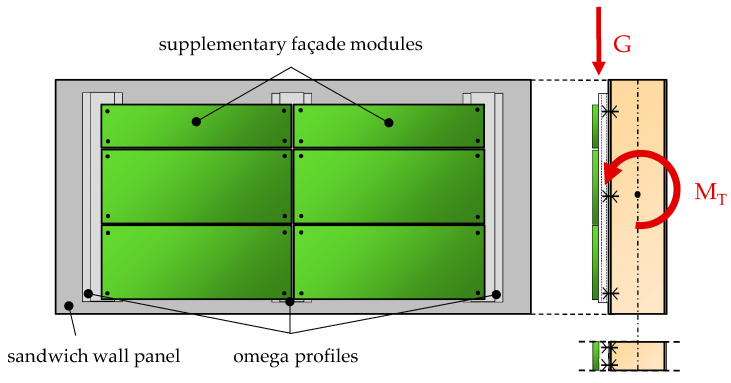
Sandwich wall panel with supplementary façade modules (schematic sketch).

In recent developments, sandwich wall panels are subjected to eccentric loads in addition to the well-known bending loads. For instance, sandwich panels equipped with photovoltaic or supplemental curtain wall modules have gained popularity [1,2]. The latter represents a novel approach that offers cost-effective and visually appealing façade solutions for both new constructions and building renovations. Façade modules of diverse architectural design can be fastened to the exterior face of the sandwich wall panels using rails (e.g., omega profiles), as shown in Figure 2. In this context, sandwich panels are the primary load-bearing component of the façade. In cases where horizontally oriented sandwich panels are used, the eccentric loads, arising from the dead weight of the additional façade modules (G), induces a torsional moment (M_T_) and resulting stresses. In summary, it is evident that with the increase in eccentricity or weight of the attached modules, the magnitude of the torsional load experienced by the sandwich panel increases too.

Previous calculation models of eccentrically loaded sandwich panels [3,4,5,6,7,8,9,10] have primarily relied on Saint-Venant’s torsion theory. However, recent research [11,12,13,14,15,16,17] has unveiled the presence of torsion-induced normal stresses in the face sheets. The findings indicate that additional mechanical effects, such as warping torsion, must be given significant consideration. Subsequently, a research project at TU Darmstadt has been initiated with the aim of developing a new engineering calculation approach to describe the structural behavior of eccentrically loaded sandwich panels from a fundamental static-mechanical perspective. To achieve this goal, comprehensive experimental, numerical, and analytical studies are conducted on sandwich panels of varying dimensions and material properties. These studies aim to thoroughly assess the theory of warping torsion, taking into account its limitations. This research article presents and discusses the results of the recent experimental and numerical parameter studies, with a specific focus on the analysis of the rotation and stress analysis in relation to warping torsion.

### 1.2. State of the Research and State of the Art

For sandwich structures, the topic of torsion was first explored in the mid-1950s and is continuously relevant until today. In 1956, Seide [3] derived the torsional stiffness for rectangular sandwich panels based on Saint-Venant’s theory. In this context, the torsional stiffness of the face sheets, the core, and their interdependent influence are considered. A similar approach was adopted by Cheng [4,5,6] although with the simplification that the core shear stresses parallel to the face sheet plane are negligible. The general solutions presented by Seide and Cheng, respectively, proved impractical for construction applications due to the mathematical complexity of the expressions, involving infinite series. A different approach was taken by Stamm and Witte [7] in 1974. Following the methodology commonly used for determining the torsional stiffness of thin-walled, closed cross-sections, they formulated a differential equation and found a more practicable solution. In their calculation model, they conceptually divided the core of the sandwich panel into an infinite number of vertical lamellae, each of infinitesimal width, which can deform independently of one another. This approach was justified by their assumption that core shear stresses in the face sheet plane can be neglected.

Torsion in sandwich panels gained increased practical relevance in the building industry during the 1980s, when the use of sandwich panels as cladding components became widespread. When a larger opening, such as a window, is introduced in a sandwich panel, it results in an eccentric load to the adjacent panel connected through a joint. This, in return, leads to torsional stress, unless an additional substructure is employed. In this context, Höglund [8] devised a formula for the torsional stiffness of sandwich panels while calculating sandwich constructions with variably positioned windows. The formula was validated through four experimental tests. In these calculations, Höglund utilized the second Bredt formula, which is typically applied to thin-walled closed cross-sections. An idealized cross-section for the sandwich panel was assumed, excluding 33% of the inner core area from consideration. In 2005, Böttcher [18] subsequently developed a simplified engineering model for an assembly of panels.

While the extensive number of theoretical publications might suggest that this is a well-explored subject, the above findings were questioned by Rädel [10,11] in the 2010s. During her research on sandwich panels with openings, she conducted two-panel tests that revealed the existence of normal stresses resulting from torsional loads. This discovery fundamentally contradicted the Saint-Venant-based torsion theories for sandwich panels mentioned earlier. According to conventional definitions, no torsion-induced normal stresses can hereby occur. Moreover, she demonstrated that the calculated torsional stiffness significantly exceeded the experimentally determined values by a factor of 1.4 to 1.6 (in comparison to Stamm and Witte’s approach) or even 6 to 10 (in comparison to Seide’s simplified approach).

In response to these findings, Pozorski and Wojciechowski [12,13,16] addressed this issue by incorporating the consideration of warping torsion. They derived a first approach of an analytical beam model for sandwich panels subjected to torsion, similar to the formulas commonly used in steel construction for straight bars with open cross-sections. Experimental and numerical studies have verified this in principle for the analyzed cases, while high attention was paid to the boundary conditions, including 1D-, 2D-, and 3D-scenarios. In addition, they noted that the more sophisticated general solution in Seide’s torsional stiffness calculation matches that of Stamm and Witte.

For additionally incorporating local effects caused by the soft core, Elmalich and Rabinovitch [19] in 2014 and Wurf et al. [17] in 2022 developed individual analytical models based on the extended high-order sandwich panel theory. Wurf et al. demonstrated favorable agreement between their calculated results and a 3D numerical solution in ANSYS for a selected example, utilizing the approach from Pozorski and Wojciechowski [13] as a reference. In detail, the results of their new approach aligned more closely with their finite element (FE) model than those shown in [13]. Nevertheless, the question remains whether the additional computational effort is justified for these minor deviations, in terms of practical applications.

From an industrial standpoint, the need to understand and account for eccentric loads in sandwich panels has grown in importance. In recent years, a rising number of manufacturers have sought European Technical Approvals (ETAs) for their sandwich panel systems, particularly those incorporating additional features such as façades or photovoltaic modules. In typical applications involving sandwich panels, the impact of torsion has historically been downplayed due to its usually negligible effect, even when dealing with minor load eccentricities. However, the increasing demand for improved thermal insulation has highlighted the influence of eccentricity on torsional loading, particularly as core thicknesses continue to increase.

Nonetheless, the current European harmonized standard for sandwich panels, EN 14509:2013 [20], does not address torsion. On a regulatory level, the CIB 378 [21] briefly mentions torsional load calculations concerning sandwich panels with openings but does not extensively consider warping torsion. Current efforts are underway to develop a new ECCS recommendation on sandwich panels [22], which will cover the effects of point and line loads, including torsion.

### 1.3. Fundamentals

In the example illustrated in Figure 2, the sandwich panel is subjected to eccentric loads due to the additional façade modules, and it can simultaneously also experience out-of-plane bending loads from wind or temperature. Numerous well-established publications [7,9,23] and the standard EN 14509:2013 offer practical calculation formulas for determining stiffnesses, deformations, and stresses under bending loads based on the First-Order Shear Deformation Theory. A distinctive feature, compared to the theory of shear-stiff beams, is that the deformations arise from both bending and shear components. In these references, sandwich panels are typically treated as beams for modelling as well as analysis, despite their plate-like appearance. In the case of torsion, this simplification is also to be initially assumed in this article. This serves as a crucial step in gaining an understanding of the structural behavior. Consequently, the term “torsion” is employed here in analogy to beams when addressing eccentrically loaded sandwich panels.

Torsion in a beam occurs when rotations, denoted as ϑ(x), appear around its longitudinal axis x, and these rotations vary along its length, indicating that the beam undergoes rotation with increasing torsion ϑ′(x) > 0. In general, torsion can be divided into Saint-Venant’s torsion (type I, free torsion) where shear stresses τ_xy_ are the primary concern, and warping torsion (type II, Vlasow torsion), in which additionally normal stresses, known as warping normal stresses σ_w_, emerge. In the field of structural engineering, the analysis typically assumes the Bernoulli and Wagner hypotheses. This means that the beams’ axis remains straight, rotations ϑ(x) occur solely around the longitudinal axis, and no cross-sectional deformations take place in the loaded state. Furthermore, influences of secondary shear deformations are neglected. Additionally, in the case of Saint-Venant’s torsion, it is presumed that warping ω or deformations out of the cross-sectional plane occur without constraint. The general differential equation for torsion is well-established. It can be expressed by the relationship between total torsional moment M_T_ and the derivations of the beam’s rotation, while introducing the decay factor λ or the structural parameter ε; see Equations (1)–(3).

(1) $$\frac{M_{T}\left(x\right)}{{\mathrm{EI}}_{W}}={-\lambda }^{2}\;\vartheta^{\prime }\left(x\right)+\vartheta^{\prime \prime \prime }\left(x\right)$$

(2) $$M_{T}\left(x\right)=M_{T,I}\left(x\right)+M_{T,\mathrm{II}}\left(x\right)$$

(3) $$\lambda =\sqrt{\frac{{\mathrm{GI}}_{T}}{{\mathrm{EI}}_{W}}}=\frac{\varepsilon }{L}$$

**Figure 3:**
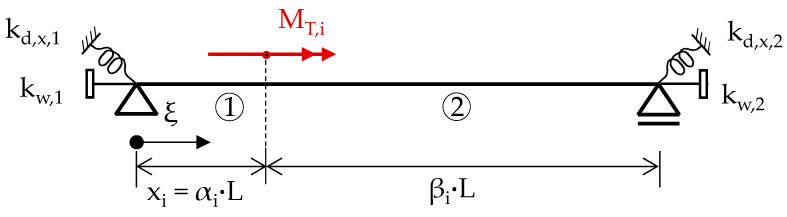
Static system of a beam subjected to a singular torsional moment.

A multitude of solutions for this differential equation, considering various boundary conditions and load applications, can be found in [24]. However, this article primarily focuses on a single span beam experiencing singular torsional moments M_T,i_ applied to an arbitrary location $x_{i}=\alpha \;\cdot L$ , while m_T_ equals zero, as illustrated in Figure 3. Recognizing the significance of boundary conditions [13,25], this article introduces both warping (k_w_) and torsional springs (k_d,x_) to the static system.

**Figure 4:**
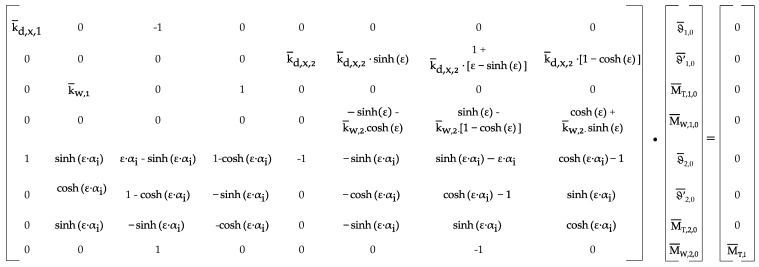
System of equations for a torsional loaded beam with warping and torsional springs.

The rotation $\vartheta \left(\xi =x/L\right)$ can be determined following the solution of the system of equations as presented in Figure 4, using Equation (4), which includes the auxiliary parameters denoted as $\bar{X}$ see Equation (5).

(4) $$\vartheta \left(\xi \right)=\bar{\vartheta }\left(\xi \right)={\bar{\vartheta }}_{j,0}+{\bar{\vartheta \prime }}_{j,0}\sinh\left(\varepsilon \;\xi \right)+{\bar{M}}_{T,j,0}\;\left[\varepsilon \;\xi -\sinh\left(\varepsilon \;\xi \right)\right]+{\bar{M}}_{W,j,0}\;\left[1-\cosh\left(\varepsilon \;\xi \right)\right]$$

(5) $$\;\;\;\;\;\bar{\vartheta }=\vartheta \;\;\;\;\;\;\;\;\;\;\;\;\;\;\;\;\;\;\;\;\;\;{\bar{M}}_{T}=\frac{M_{T}}{{\mathrm{GI}}_{T}}\cdot \left(\frac{L}{\varepsilon }\right)\;\;\;\;\;\;\;\;\;\;\;\;\;\;\;\;\;\;\;\;\;\;\;\;\;\;\;\;{\bar{k}}_{d,x}=\frac{k_{d,x}}{{\mathrm{GI}}_{T}}\cdot \left(\frac{L}{\varepsilon }\right)\;{\bar{\vartheta }}^{\prime }=\vartheta^{\prime }\cdot \left(\frac{L}{\varepsilon }\right)\;\;\;\;\;\;\;\;\;\;\;\;\;\;\;\;{\bar{M}}_{W}=\frac{M_{W}}{{\mathrm{GI}}_{T}}\;\;\;\;\;\;\;\;\;\;\;\;\;\;\;\;\;\;\;\;\;\;\;\;\;\;\;\;\;\;\;\;\;\;\;\;{\bar{k}}_{w}=\frac{k_{w}}{{\mathrm{GI}}_{T}}\left(\frac{\varepsilon }{L}\right)\;$$

Before these universal equations and methods can be employed for sandwich panels, it is necessary to establish the panels stiffness. In line with the current state of research [11,13,16,17], the following stiffness values are considered appropriate, as proposed by Stamm and Witte [7]. They introduced the torsional stiffness denoted as ${\mathrm{GI}}_{T}$ as follows.

(6) $${\mathrm{GI}}_{T}=4\;G_{F}{\;e}^{2\;}b\;\frac{t_{1}t_{2}}{t_{1}{+t}_{2}}\left(1\;-\;\frac{\tanh(\Omega b/2)}{\Omega b/2}\right)$$

(7) $$\Omega =\frac{G_{\mathrm{xz},C}}{G_{F}}\frac{t_{1}{+t}_{2}}{d_{C}\cdot {\;t}_{1}t_{2}}$$

Applying the warping torsion theory on a sandwich panel in analogy to an I-beam, the following Equation (8) is derived.

(8) $${\mathrm{EI}}_{W}=\frac{E_{F}{\;b}^{3}}{12}\;\left({e_{1}}^{2}{\;t}_{1}{{+e}_{2}}^{2}{\;t}_{2}\right)$$

The torsion-induced stresses in the core and face sheets of the sandwich panel can be computed using the following equations for both xz- and xy-stress components. Here, it should be noticed that, in contrast to the shear stress distribution of a torsion-loaded I-beam, the primary shear stress in the “flanges” (here: face sheets) is not uniformly distributed along the panel width but is hyperbolic with a peak at mid-width.

(9) $$\tau_{\mathrm{xz},I,C}=\frac{\Omega \;\sinh(\Omega \;y)}{\cosh(\Omega \;b/2)-\frac{\sinh(\Omega \;b/2)}{\Omega \;b/2}}\;\frac{M_{T,I}}{2\;b\;e}$$

(10) $$\tau_{\mathrm{xy},\;I,F}=\frac{\cosh\left(\Omega b/2\right)-\cosh(\Omega \;y)}{\cosh(\Omega \;b/2)-\frac{\sinh(\Omega \;b/2)}{\Omega \;b/2}}\;\frac{M_{T,I}}{{2\;b\;e\;t}_{1,2}}$$

(11) $$\tau_{\mathrm{xy},\mathrm{II},F}=\frac{M_{T,\mathrm{II}}{\;S}_{w}}{I_{W}{\;t}_{1,2}}$$

(12) $$\sigma_{w,F}=\frac{M_{W}}{I_{W}{\;t}_{1,2}}{\bar{\omega }}^{M}$$

## 2. Parametric Analysis Methods for Eccentrically Loaded Sandwich Panels

### 2.1. General

To comprehensively assess warping torsion in sandwich panels, a series of experimental and numerical parameter studies was conducted. PU foam core sandwich panels with quasi-planar face sheets, commonly employed in European building envelopes, are the subjects of these studies. Various dimensions and material properties were taken into account, while longitudinal joints were neglected. The primary focus of these studies is directed towards the structural response, particularly in terms of rotation, as well as normal and shear stresses in the face sheets and core.

The experimental study supplied crucial data on actual sandwich panels used in construction and permits the validation of the numerical model in ANSYS. In contrast, the numerical study, based on a calibrated model, provides a valuable advantage by enhancing the experimental scope and offering deeper insights into aspects that may not be readily determined through direct measurements.

### 2.2. Experimental Study

#### 2.2.1. Test Setup and Measurements

**Figure 5:**
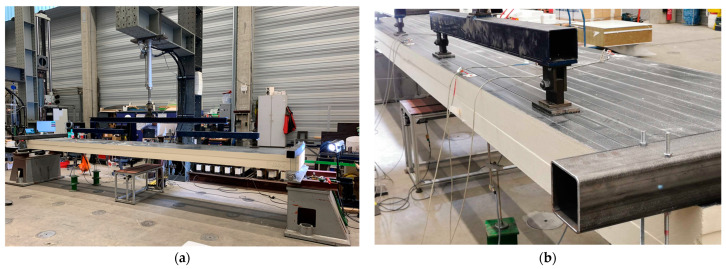
Eccentric 6-point bending test setup: (**a**) full view; (**b**) lateral perspective.

As part of the experimental studies, a newly developed eccentric 6-point bending test was conducted on several PU sandwich panels from two different manufacturers. This test setup is primarily derived from the standardized test used to assess the load-bearing capacity of sandwich panels in accordance with EN 14509:2013, Annex A. The significant modifications to the conventional test are elaborated upon in the following and can also be referenced in Figure 5.

- In addition to the bending loads, a torsional moment is induced by the eccentric application (e = 350 mm) of four-point loads.
- The point loads are applied to the top face sheet using a steel plate with the dimensions 100 mm × 150 mm × 10 mm and an elastomer layer underneath.
- The pin support on both sides is designed based on a fork support.
- The maximum load was set at 50% of the experimentally determined ultimate load for the centrically loaded reference specimen, ensuring that the tested materials remain within the linear-elastic range. This reference specimen was exclusively subjected to bending following EN 14509:2013, A.5.

**Figure 6:**
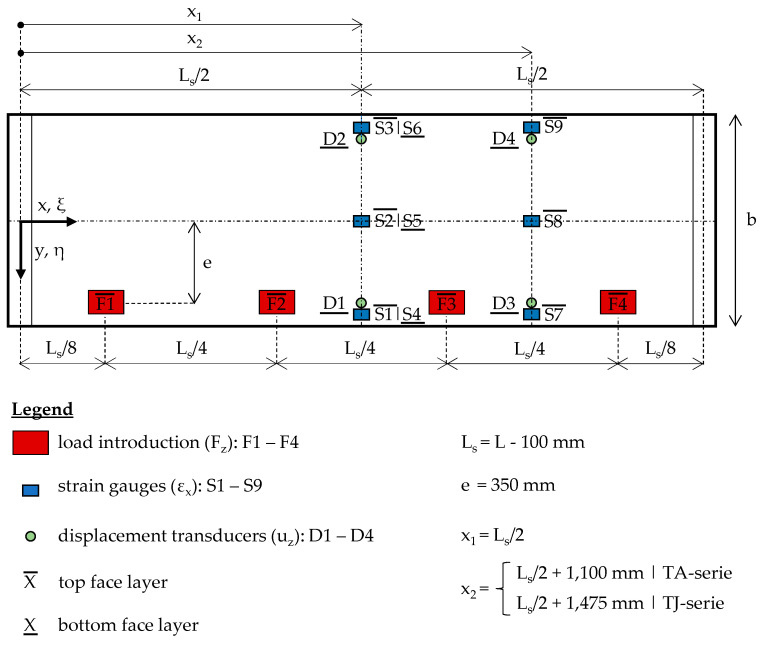
Test setup in top view and arrangement of the basic measurement.

Figure 6 illustrates the test setup schematically in the top view. Here, the arrangement of the basic measurements carried out for all test specimens are shown. In total, nine strain gauges (S1–S9) were applied on both the top and the bottom face sheets, positioned at midspan (x_1_) and between the third and fourth load introduction points (x_2_). Additionally, four displacement transducers (D1–D4) were positioned at x_1_ and x_2_ to assess the vertical displacement of the bottom face sheet. Supplementary measurements were also taken. However, they are not exhaustively discussed in this article.

#### 2.2.2. Fork Support

In the context of the eccentric 6-point bending test, the utilization of a fork support on both sides proves beneficial for investigating the structural response of sandwich panels under torsion. However, achieving an ideal fork support in practical applications, especially for sandwich panels, composed of materials with significantly different stiffnesses, is a challenging endeavor. The primary aim was to develop a support system that fulfills the following requirements: ideally preventing rotations caused by a torsional moment (M_T_), allowing warping to a certain extent, and remaining hinged concerning bending moments (M_y_).

**Figure 7:**
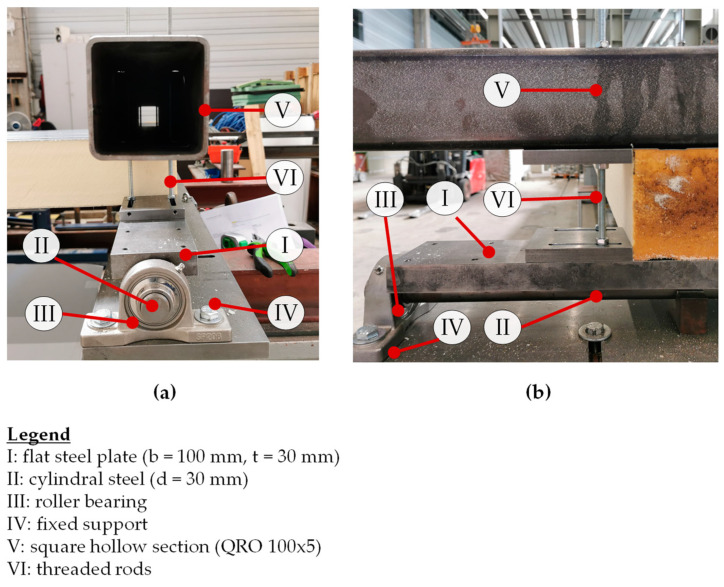
Newly developed bearing construction: (**a**) side view; (**b**) front view.

As depicted in Figure 7, the newly devised bearing construction represents an optimized iteration of the one utilized in a previous study [14]. The sandwich panels are positioned upon a flat steel plate (I) that is welded to a cylindrical steel element (II) supported by a roller bearing (III), enabling it to rotate with minimal friction. The roller bearings are securely attached to the substructure through a fixed support (IV). To restrict deformations of the cylindrical steel, three semi-circular plain bearings are symmetrically arranged beneath it. At the top of the sandwich panel, a square hollow section (V) is positioned and secured to the flat steel using threaded rods (VI) to prevent rotation around the longitudinal axis of the panel.

#### 2.2.3. Test Program

**Figure 8:**
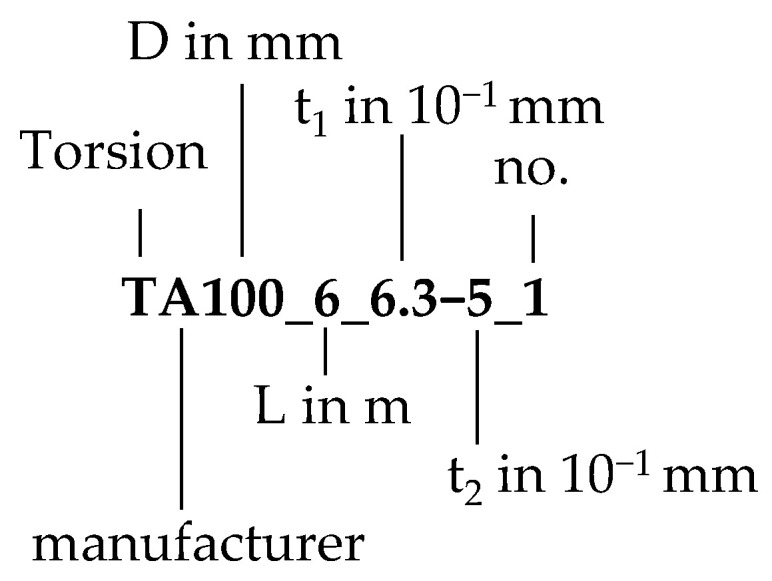
Definition of the configuration.

During the experimental study, a total of 20 distinct configurations were tested. In this context, a configuration is defined as the specific parameter set of the investigated sandwich panels, see Figure 8. The analyzed parameters include the total length L, the core thickness D, and the face sheet thickness t_1_ and t_2_, respectively. Each configuration was assessed with up to three specimens, resulting in a total number of 52 tests. The parameter ranges for these configurations are listed in Table 1.

To ensure comparability, a uniform total width of b = 900 mm was maintained across all the tested sandwich panels. In fact, the geometry and design of the longitudinal joints significantly varies depending on the manufacturer and the core thickness. Therefore, these joints were symmetrically removed from both sides before the tests. The width indicated here refers to the remaining width (b = 900 mm).

#### 2.2.4. Material Tests

The material properties of the PU core sandwich panels under examination were tested at room temperature in accordance with EN 14509:2013, Annex A. Table 2 presents a summary of the value ranges for the shear and Young’s modulus of the specimen.

### 2.3. Numerical Study

**Figure 9:**
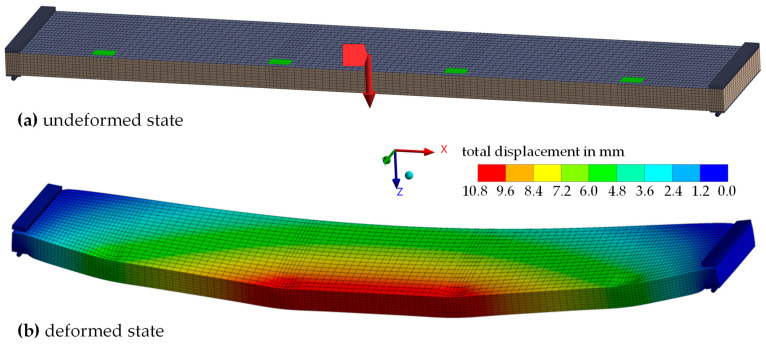
Numerical model in ANSYS Workbench 2022 R2, exemplary for the configuration TA160_5-6.3-5: (**a**) in the undeformed state; (**b**) in the deformed state (F = 3 kN).

For the numerical investigation, a parametric 3D FE model of the tests described in Section 2.2. was developed in ANSYS Workbench 2022 R2 [26], as shown in Figure 9. In this model, the face sheets were idealized as plane surfaces using 4-noded shell elements (SHELL281), while the core was modelled with 8-noded solid elements (SOLID185). Similar solid elements were employed for the bearing structure, except for the threaded rods, for which BEAM188 elements were chosen.

The steel face sheets of the sandwich panel and all other steel components were characterized using an isotropic linear elastic material model with E_F_ = 210,000 MPa and µ_F_ = 0.3. The PU foam sandwich core was defined as an anisotropic linear elastic material with shear and Young’s moduli set equally in all directions, and a Poisson’s ratio µ_C_ of 0.25, according to [13,18,23]. The mesh size was specified at 25 mm on average.

Boundary conditions were established for the roller bearings by defining a general “Body-Ground” connection, fixing the relevant translations and rotations. Since the face sheets and core were treated as one component in the ANSYS tool DesignModeler there was no need to explicitly define contact within the sandwich panel. In contrast, contact elements between the sandwich panel and the bearing structure were manually defined as CONTA174 and TARGE170, respectively. For both the upper and lower interfaces, three types of contact were considered: bonded, rough, and frictional. The contact types differ in the forces they transfer. Bonded contact transmits compressive, tensile, and shear forces, whereas the non-linear contacts (rough and frictional) exclude tensile forces but transmit shear and compressive forces. In the frictional type, shear forces are transmitted based on the friction value. For the non-linear contact types, the “Large Deflection” setting is configured as recommended, considering the effects from second-order theory [26].

The numerical study is divided into two distinct parts, with technical data concerning material and geometrical being categorized accordingly.

- In the validation of the numerical model, a frictional contact type was selected and the experimentally determined material properties and dimensions were employed, as detailed in Table 1 and Table 2.
- In the subsequent extended numerical investigations, a rough contact type was chosen. Table 3 provides information regarding the configuration of the reference models for the extended numerical study and the range of the analyzed parameters for potential practical use cases. The values specified for the elastic and shear moduli apply uniformly in all three spatial directions unless otherwise specified.

**Figure 10:**
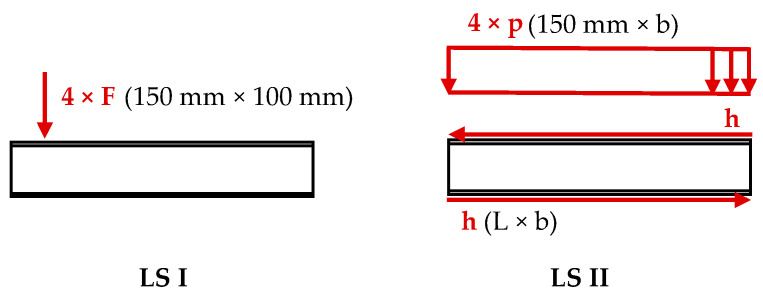
Load situations I (6-point bending tests) and II (superposition of bending and torsion) illustrated on the cross-section of a sandwich panel. In parentheses, the area on which the individual surface loads are applied.

Furthermore, two load situations (LS) were considered for the simulation with respect to the extended numerical studies. They involved uniformly distributed surface loads with deformable behavior acting on a defined area (indicated in parentheses). Primarily, in LS I, four vertical eccentric surface loads F were applied (150 × 100 mm), similar to the eccentric 6-point bending test. Secondly, in LS II, a superposition of “pure” bending and “pure” torsion loads was introduced. For the bending part, four vertical centric surface loads p (150 mm × b) were applied, and for the torsional part, opposite-directed horizontal surface loads (L × b) in the plane of the face sheets were introduced, see Figure 10. Both load situations were applied in a single time step each.

### 2.4. Analytical Study

In this article, the analytical study serves the specific purpose of establishing relationships between the outcomes of the experimental and numerical methods, particularly with regard to warping torsion. For the warping torsion theory calculations, formulas described in Section 1.3 were applied with and without torsional and warping springs.

In the consideration of springs, a test-based best-fit method was employed, using the Method of Least Squares and adopting the basic approach outlined in [27]. This approach aimed to determine the spring values k_d,x_ and k_w_ for each tested or simulated configuration of sandwich panels. If not explicitly mentioned otherwise, this case was applied in this article when referring to the analytical approach. In the alternative case of neglecting at least one spring type, $k_{w}\to \;0$ or $k_{d,x}\to \;\infty$ was applied, respectively.

The superposition principle was applied concerning both bending and torsion, as well as the four-point load, in accordance with the initial assumption that testing and simulation occurred within the linear elastic range of the sandwich panels. For the bending behavior, the relevant equations for the displacement and stress analysis of the face sheets and the core of the sandwich panels were utilized from [7].

## 3. Results from Experimental and Numerical Studies Considering Warping Torsion

### 3.1. General

First, representative experimental results are shown in conjunction with the numerical and analytical solutions. Second, the results of the extended numerical study beyond the scope of the experiments are provided.

### 3.2. Experimental Results and Validation of the Numerical Model

**Figure 11:**
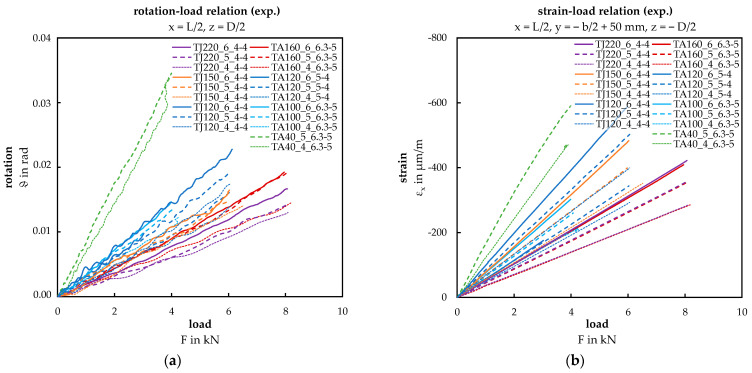
Load diagrams: (**a**) rotation–load relation; (**b**) strain–load relation.

Figure 11 shows the experimentally determined rotation–load (a) and the strain–load (b) relationships for each tested configuration. In both diagrams, a clear, nearly linear association is observed between the applied load and the rotation or strain at midspan. The rotation ϑ is calculated under the assumption of an idealized, linearized deformation of the face sheets across the width of the panel. The value u_z_ represents the displacement transducers, and c signifies the distance between these measurement points.

(13) $$\vartheta \;\left(x_{1}\right)=\frac{u_{z,\;D1}-{\;u}_{z,D2}}{c}$$

The measured value of the strain gauge S3, located at x = L/2, y = −b/2 + 50 mm, z = −D/2 is expressed in µm/m. In the following, the resulting normal stress is provided in MPa by multiplying these strain values with the modulus of elasticity of E = 210,000 MPa.

**Figure 12:**
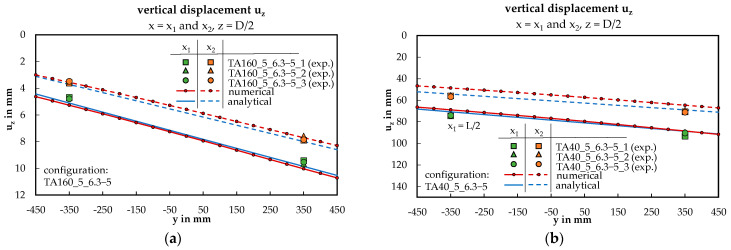
Rotation of the panel and deflection of the bottom face sheet across the panel width at x_1_ and x_2_, shown as exemplary for two configurations: (**a**) TA160_5_6.3-5; (**b**) TA40_5_6.3-5.

**Figure 13:**
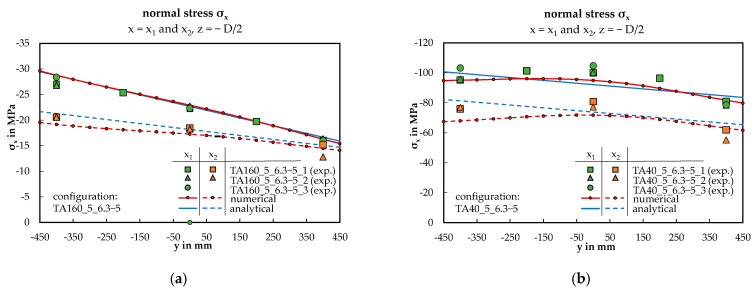
Normal stress of the top face sheet across the panel width at x_1_ and x_2_, shown as exemplary for two configurations: (**a**) TA160_5_6.3-5; (**b**) TA40_5_6.3-5.

In the following four diagrams in Figure 12 and Figure 13, the focus is on configurations TA160_5_6.3-5 and TA40_5_6.3-5 with an applied load of F = 3 kN. Figure 12 displays the deflection curves of the bottom face sheet at points x_1_ (midspan) and x_2_ (between the third and fourth load introduction). The data points obtained from displacement transducers D1 to D4 closely align with the red and blue curves, indicating good agreement.

Figure 13 displays the distribution of normal stress in the top face sheet across the width of the panel at points x_1_ and x_2_. In Figure 13a, a nearly linear curve is evident, while in Figure 13b, it exhibits non-linearity. In both cases, the numerical curves closely match the experimental data points, which is not the case for the analytical approach of part (b). Similar results have been obtained for the bottom face sheet but are not further discussed.

**Figure 14:**
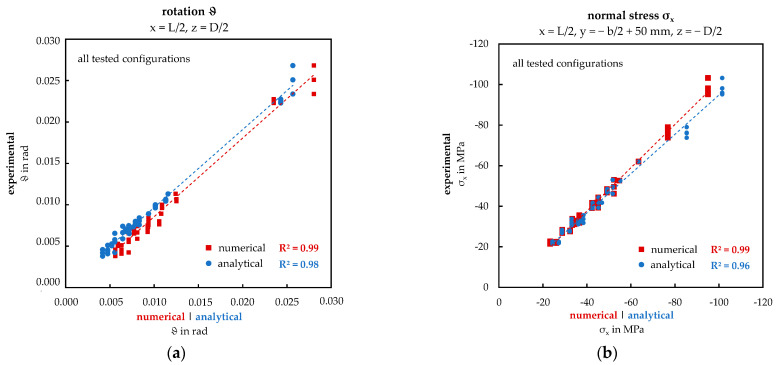
Scatter plots of the experimental, numerical, and analytical results: (**a**) rotation ϑ (x = L/2, z = D/2); (**b**) normal stress σ_x_ (x = L/2, y = −b/2 + 50 mm, z = −D/2).

To validate and quantify the presented qualitative alignment between the experimental and numerical approach, scatter plots including coefficient of determination R^2^ are introduced, including all 20 tested and simulated sandwich panel configurations concerning the rotation and the normal stress at midspan, see Figure 14. For both magnitudes, the R^2^ value is high (>0.95), which proves there is a strong correlation between the approaches across the wide range of considered parameters and supports the qualitative compliance shown above. At the same time, the numerical model is validated. In addition, the result of the analytical calculation shows a comparably high correlation with the other two approaches.

### 3.3. Further Numerical Studies

In the experimental study, practicable constraints may limit the ability to measure all points of interest in detail. Consequently, specific points are selectively analyzed and assessed in the numerical approach. Furthermore, significant insights can be extracted from the numerical parametric study to break down the fundamental structural behavior of eccentrically loaded sandwich panels into specific parameters or parameter ratios. This process facilitates identifying potential expressions for engineering calculation methods.

#### 3.3.1. Shear Stress Analysis

**Figure 15:**
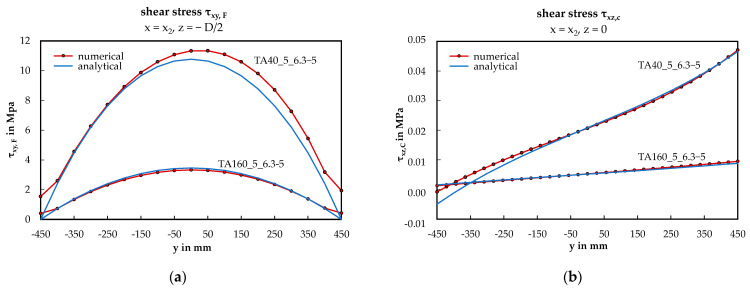
Shear stress distribution across the panel width: (**a**) shear stress of the face sheets (x = x_2_, z = − D/2); (**b**) shear stress of the core (x = x_2_, z = 0).

To highlight the shear stress distribution, the numerical results of the two representative configurations TA160_5_6.3-5 and TA40_5_6.3-5 are shown in relation to the analytical solutions in Figure 15. On the right side (b), the stresses in the middle of the core were calculated by superimposing the vertical force and torsion. For the vertical force, a uniformly distributed core shear stress across the panel width was assumed, neglecting the effects of local load introduction.

#### 3.3.2. Extended Numerical Parametric Studies

Through the extended numerical parametric study based on the reference model (see Table 3), incorporating 48 configurations, important relations between the outcome variables (rotation, stress) and the geometric input variables were found and compared to the analytical approach based on warping torsion.

**Figure 16:**
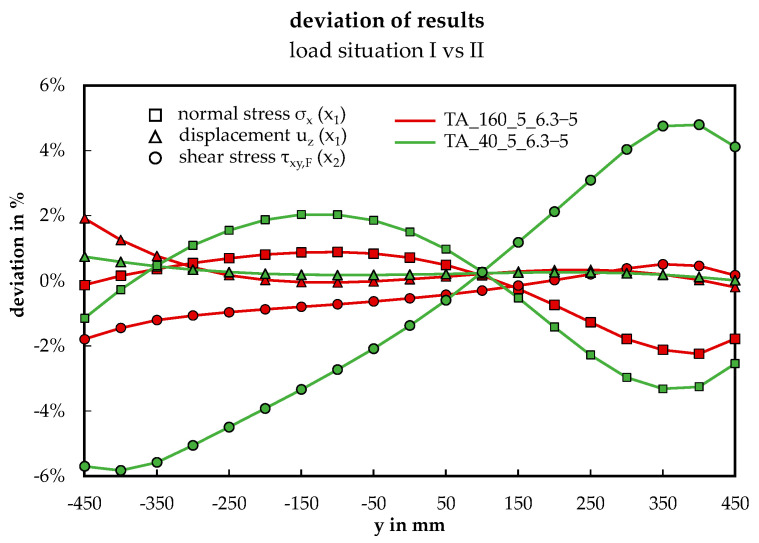
Deviation of results (σ_x_, u_z_, τ_xy_) of the load situations I and II.

While conducting experiments, achieving a “pure” torsional loading on sandwich panels is not practicable. However, for the subsequent extensive numerical investigation, a more effective understanding of the structural response to pure torsion can be attained by decomposing the bending and torsion component from the applied eccentric four-point-load, as described in load situation LS II. Figure 16 illustrates that this approach is acceptable for the present investigation. The deviation of the results σ_x_ (x_1_), u_z_ (x_1_), τ_xy,f_ (x_2_) obtained from the validated numerical model applying LS I and LS II is low (< 6%), presumably resulting from the concentrated load introduction, see [28].

Using the validated FE model, the parameter window of the experimental parameter study (see Table 1 and Table 2) is comprehensively expanded regarding the geometry of the panels (see Table 3). The numerical solution was categorized using the three analytical approaches considering complete (blue), none (violet), and partial warping restraint (green). Through a test-based best-fit method mentioned in Section 2.4, the torsion spring constant, here the value k_dx_ = 0.79 was determined for the described six-point bending test setup. However, fitting not only k_dx_ but also k_w_, and both values separately for each tested sandwich panel configuration, leads to the green data points.

**Figure 17:**
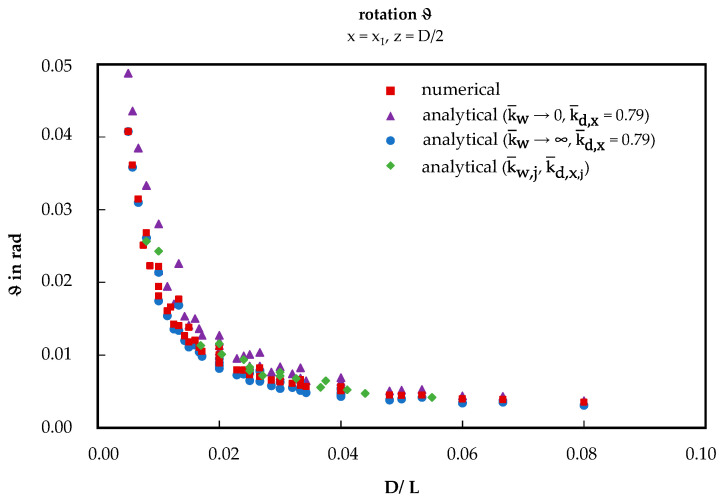
Rotation dependent on geometric ratios.

Plotting the rotation over the ratio D/L reveals a hyperbolic relationship between these quantities (see Figure 17). The numerical results align between both analytically determined data points with and without warping restraint across the entire spectrum. This also applies to the configuration-dependent best-fit approach.

**Figure 18:**
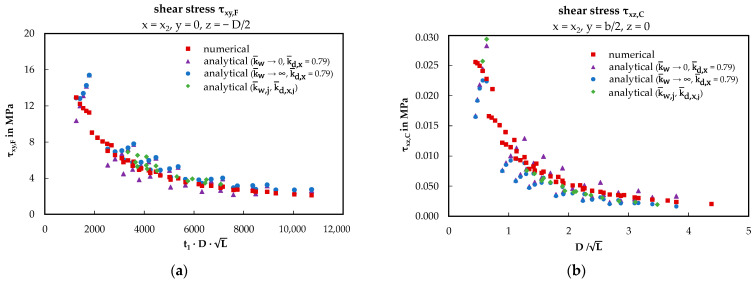
Shear stress dependent on geometric ratios: (**a**) shear stress of the face sheets (x = x_2_, y = 0, z = − D/2); (**b**) torsion-induced shear stress of the core (x = x_2_, y = − b/2, z = 0).

Similar observations are made regarding the shear stresses in Figure 18, both in the face sheets (a) and in the core (b). In the left case (a), there is a hyperbolic relationship between shear stress and the product of the sheet thickness t_1_, the core thickness D, and the square root of the component length L. In the right case (b), there is a hyperbolic relationship between the torsional induced core shear stress and the quotient of D and the square root of L. It is noteworthy that the green data points follow the red ones despite different initial parameters.

## 4. Discussion on Experimental and Numerical Results considering Warping Torsion

### 4.1. General

Throughout the comprehensive studies, a consistency in the results obtained from the experimental, numerical, and analytical methods was observed. In detail, the findings will be discussed with regard to the assumptions, calculation models, and possible limitations of the methods.

### 4.2. Rotation of the Sandwich Panel

The rotation of the sandwich panels was calculated using vertical displacement, assuming a linear distribution across the panel width. This assumption was validated as the deflection exhibited linearity across the width for both examined configurations. The displacement curves TA160_5_6.3-5 and TA40_5_6.3-5 are nearly identical for both considered locations, x_1_ and x_2_, across all three methods. Correspondingly, the derived rotation values from this analysis are also in agreement as demonstrated across the entire scope of tested configurations in the scatter plot presented in Figure 14a (R^2^ > 0.98). Residuals at high rotation values can be attributed to fluctuations of the material properties or inaccurate measurements. In the extended numerical study, a hyperbolic relationship between the rotation and the ratio D/L was identified (see Figure 17). In this context, the numerical solution aligns closely with the analytical results, considering full, none, or partial warping restraint. Notably, the configuration-dependent test-based best-fit method exhibits the most accurate alignment with the numerical solution.

### 4.3. Normal Stresses in the Face Sheets

According to classical warping torsion theory, linear stress distributions in the flanges (in this case: face sheets) are anticipated. Unlike an eccentrically loaded homogeneous plate, the maximum normal stresses are expected to be localized on the load-averted side. Both statements are generally confirmed for the presented configuration TA160_5_6.3-5 across all three methods, particularly for x_1_, as illustrated in Figure 13a. However, for TA40_5_6.3-5, featuring the lowest examined core thickness of D = 40 mm, qualitative and quantitative deviations are apparent; see Figure 13b. While the numerical solution aligns with the non-linear distribution obtained from the experimental data points, the analytical approach, by definition, leads to a linear curve. Notably, considering that non-linearity is more prominent on the load-averted side (−b/2 ≤ y < 0), the linear approach appears to remain suitable for the load-facing side (0 < y ≤ b/2). Influences resulting from the boundary conditions or the geometrically non-linear load effects may contribute to favoring this non-linearity. Considerations of these factors were integrated into the FE model but were not comprehensively addressed in the analytical model of warping torsion.

In the finite element model, frictional contact definitions between the face sheet and the bearing structure capture the normal stress gradient more accurately than other considered contact definitions. The friction value, calibrated to 0.1, accounts for the effective area activated for friction (considering the presence of lined face sheets) and the characteristics of the material surfaces. The newly designed fork support may exhibit clearance due to the compressibility of the core and stiffness in lateral bending. Analytically, this effect was considered only through the introduction of the torsional and warping spring for both support conditions using the test-based best-fit method.

In the ANSYS setting “Large Deflection”, effects from second-order theory were considered. However, this aspect seems contradictory to the experimentally determined displacement–load and strain–load relationship (see Figure 11), assumed to be nearly linear for the applied load range.

Conversely, for core thicknesses above D = 40 mm, the significance of the contact definition and the second-order theory (“Large Deflection”) gradually decreases for the tested configurations. Preliminary numerical studies on sandwich panels with thicker core thicknesses, neglecting these assumptions, have already indicated this. This trend is evident in the scatter plot (Figure 14b), which also highlights the high correlation between the different approaches (R^2^ > 0.98). It is presumed that in sandwich panels with low core thicknesses, a secondary structural mechanism occurs in addition to the warping torsion.

### 4.4. Shear Stresses in the Face Sheets and in the Core

The shear stress analysis is conducted based on the analytical and the validated numerical model. For both depicted shear stresses (see Figure 15), observed in the top face sheets and in the middle of the core, the numerical and analytical solutions exhibit perfect alignment for the configuration TA160_5_6.3-5. Deviations, however, occur for TA40_5_6.3-5. Regarding the shear stress τ_xy,F_, a slight asymmetry is evident, with both lines coinciding on the load-averted side, excluding the fact that the numerical solution does not approach zero in the edge. In contrast, for the shear stress τ_xz,C_, both solutions align on the load-faced side but diverge on the load-averted side. Presumably, both phenomena are linked to the effects of the local load introduction. It is noteworthy that the value from the torsional induced shear stress in the core is comparable to the value from the vertical load component. Nevertheless, the analytical formula for determining the shear stress can be confirmed in principle.

Similar to the rotation analysis, the extended numerical study reveals hyperbolic relationships between the considered magnitudes and geometric ratios. For the shear stress (see Figure 18) in the face sheets, the abscissa is the product of the core thickness, the square root of the total length and the sheet thickness. Meanwhile, the abscissa for the core shear stress is represented by the quotient of core thickness and the square root of the total length. Once again, the test-based best-fit approach most accurately aligns with the numerical solution, capturing effects beyond the purely analytical approach.

### 4.5. Superposition Principle of Bending and Torsion in Sandwich Panels

The presented displacement and stress curves confirm that bending and torsion can be superimposed to represent eccentric loads for sandwich panels, despite their plate-like appearance. Furthermore, the small deviations between the load situations I (six-point bending test) and II (superposition of bending and torsion), as introduced in Figure 10 and illustrated in Figure 16, indicate additionally the influence of the local load introduction concerning the transversal direction is recognizable but small.

## 5. Conclusions, Contribution, and Future Work

In this research article, the structural behavior of eccentrically loaded sandwich panels with a PU foam core was investigated through a combination of experimental and numerical studies and was compared to the analytical approach based on warping torsion. For this purpose, a newly developed eccentric six-point single-span bending test was introduced with the primary goal of gathering essential data on rotation and normal stress values for various sandwich panels with different geometries and material properties. Subsequently, a numerical model was developed and validated, enabling an expanded parameter study and a detailed examination regarding shear stresses. The key findings and contributions from this study are summarized as follows:

- A high correlation was observed between experimental, numerical, and analytical results for 20 different parameter configurations, particularly concerning the rotation and normal stress values at the midspan of the sandwich panels.
- The qualitative curve of the stress and displacement distribution across the width of the panel are in general aligned for all three methodologies, in particular for the tested sandwich panels with a core thickness higher than D = 40 mm.
- The shear stress of the face sheets and the core of the sandwich panel from the analytical approach on warping torsion agrees in general with the numerical results.
- The superposition principle concerning bending and torsion loadings is confirmed for the tested configurations. Only minor deviations were observed in comparison to the eccentric loading.
- The applicability of warping torsion for PU sandwich panels has been demonstrated within a considerable practical parameter range, considering various material properties and dimensions. A test-based best-fit method based on warping torsion was applied for the analytical approach. However, it is important to acknowledge the limitations of this approach regarding non-linear stress distributions.
- From the extensive parametric study based on a validated numerical model hyperbolic relations were found between the rotation values and the ratio D/L, face shear stress and the ratio t_1_ ·  D $/\sqrt{L}$ , as well as the core shear stress and the ratio D $\cdot \sqrt{L}$ .
- The magnitude of stresses may not necessarily be negligible in terms of design considerations and can become significant.

For future research, it is recommended to address the constraints of the warping torsion theory, particularly in terms of geometric and stiffness ratios, which are evident in the non-linear stress distribution. The presented numerical approach is well-suited to conduct further detailed studies with the aim of finding a practicable engineering calculation model. While it may involve a more complex mathematical process, it is suggested to explore alternative analytical methods, such as the sandwich plate theory [7], for calculating eccentrically loaded sandwich panels. Mechanically complex, but not considered in this article, influences from secondary shear deformation could be relevant [11].

Nevertheless, the primary objective should remain focused on developing a simplified analytical or semi-analytical approach based on the data obtained from the experimental and numerical studies. This approach should concentrate on the design of safe and economically efficient building envelopes, while exploring the potential to expand the scope of applications for sandwich panels.

## Figures and Tables

**Table 1:** Assessed parameter range for the eccentric 6-point bending tests.

Parameter	Symbol	Values
Total length	L	4000–6000 mm
Core thickness	D	40–220 mm
Sheet thickness	t_1,_ t_2_	0.40–0.63 mm

**Table 2:** Assessed parameter range for the eccentric 6-point bending tests.

Parameter	Symbol	Mean Values in MPa
Shear modulus	G_C,xz_	3.0–4.4
Young’s modulus (compression)	E_Cc_	3.3–5.2
Young’s modulus (tension)	E_Ct_	3.3–4.7

**Table 3:** Reference values and parameter range for the extended numerical study.

Parameter	Symbol	Reference	Parameter Range
shear modulus ^1^	G_C_	4 MPa	2–6 MPa
Young’s modulus	E_C_	4 MPa	4 MPa
Poisson’s ratio	µ_C_	0.25	0.25
core thickness	D	100 mm	40–250 mm
total width	b	900 mm	450–1350 mm
total length	L	5000 mm	3000–8000 mm
sheet thickness ^1^	t_1_, t_2_	0.5 mm	0.4–0.6 mm
total load ^1^	F	3 kN	1 kN–5 kN

^1^ In the present article, results from the extended numerical study are shown only with G_C_ = 4 MPa, t_1_ = t_2_ = 0.5 mm, and F = 3 kN.

## Data Availability

The data presented in this study are available on request from the corresponding author.
